# Shortness of breath on the day of discharge: an early alert for post-discharge complications in patients undergoing lung cancer surgery

**DOI:** 10.1186/s13019-024-02845-1

**Published:** 2024-06-27

**Authors:** Dan Kang, Cheng Lei, Yong Zhang, Xing Wei, Wei Dai, Wei Xu, Jingyu Zhang, Qingsong Yu, Xueyao Su, Yanyan Huang, Qiuling Shi

**Affiliations:** 1 School of Public Health, Chongqing Medical University, Chongqing, People’s Republic of China; 2 Department of Thoracic Surgery, Sichuan Clinical Research Center for Cancer, Sichuan Cancer Hospital & Institute, Sichuan Cancer Center, Affiliated Cancer Hospital of the University of Electronic Science and Technology of China, Chengdu, China; 3 State Key Laboratory of Ultrasound in Medicine and Engineering, College of Biomedical Engineering, Chongqing Medical University, Chongqing, People’s Republic of China; 4 Chengdu Center for Disease Control and Prevention, Chengdu, 610041 Sichuan China

**Keywords:** Lung cancer, Post-discharge complications, Patient-reported outcomes, Shortness of breath, Cutpoint

## Abstract

**Purpose:**

Symptom assessment based on patient-reported outcome (PRO) can correlate with disease severity, making it a potential tool for threshold alerts of postoperative complications. This study aimed to determine whether shortness of breath (SOB) scores on the day of discharge could predict the development of post-discharge complications in patients who underwent lung cancer surgery.

**Methods:**

Patients were from a study of a dynamic perioperative rehabilitation cohort of lung cancer patients focusing on patient-reported outcomes. Patients were assessed using the Perioperative Symptom Assessment Scale for Lung surgery (PSA-Lung). Logistic regression model was used to examine the potential association between SOB on the day of discharge and complications within 3 months after discharge. The post-discharge complications were taken as the anchor variable to determine the optimal cutpoint for SOB on the day of discharge.

**Results:**

Complications within 3 months post-discharge occurred in 71 (10.84%) of 655 patients. Logistic regression analysis revealed that being female (OR 1.764, 95% CI 1.006–3.092, *P* < 0.05) and having two chest tubes (OR 2.026, 95% CI 1.107–3.710, *P* < 0.05) were significantly associated with post-discharge complications. Additionally, the SOB score on the day of discharge (OR 1.125, 95% CI 1.012–1.250, *P* < 0.05) was a significant predictor. The optimal SOB cutpoint was 5 (on a scale of 0–10). Patients with an SOB score ≥ 5 at discharge experienced a lower quality of life 1 month later compared to those with SOB score<5 at discharge (73 [50–86] vs. 81 [65–91], *P* < 0.05).

**Conclusion:**

SOB on the day of discharge may serve as an early warning sign for the timely detection of 3 month post-discharge complications.

**Supplementary Information:**

The online version contains supplementary material available at 10.1186/s13019-024-02845-1.

## Introduction

Lung cancer ranks among the most prevalent cancers globally, with approximately 2 million new cases diagnosed each year [1, 2]. Surgical resection is the preferred treatment for patients with early-stage lung cancer and remains an option for those with intermediate and advanced stages [3]. However, lung resection can lead to postoperative complications [4, 5], which occur more frequently in patients who underwent lung cancer surgery compared to other surgical procedures, with incidence rates ranging from 14 to 40%. These complications can markedly affect patients’ quality of life, prognosis, and long-term survival [6].

Shortness of breath (SOB) is a significant symptom reported by up to 90% of patients with lung cancer during postoperative recovery following lung surgery, markedly affecting their wellbeing [7, 8]. Patients experiencing moderate-to-severe SOB are more likely to encounter adverse events and complications after surgery, with an increased risk of postoperative readmission and mortality, as indicated by Thoracoscore calculations [9]. The American Thoracic Society defines SOB as a subjective sensation of respiratory discomfort, varying in intensity and nature. It can be accurately measured by assessing the frequency and severity scores of SOB reported by patients [7], using a numeric rating scale. Wysham et al. have established thresholds for SOB severity, with 0–4 indicating none/mild and ≥ 5 signifying moderately severe SOB [10]. Similarly, a Respiratory Distress Observation Scale score of 0–3 suggests absent/mild SOB, while a score of ≥ 4 denotes moderately severe SOB [11].

The widespread adoption of fast-track protocols, such as Enhanced Recovery After Surgery Programs, coupled with economic pressures on insurers and hospitals, has led to a continued decrease in the average length of stay following surgery. Consequently, there has been an uptick in the percentage of post-discharge complications [12]. Research indicates that the incidence of complications after discharge remains substantial, particularly among postoperative general surgery patients, with approximately 32.9% of post-surgical complications occurring post-discharge [13, 14]. Evaluating SOB on the day of discharge could potentially act as an early indicator of post-discharge complications, as it is a significant predictor of such complications and subsequent readmission [15].However, it is important to acknowledge the current lack of sufficient evidence to fully endorse this practice.

Our team previously analyzed the relationship between SOB and complications during postoperative hospitalization [16]. The association between SOB and post-discharge complications remains unclear, necessitating further investigation. Thus, the current study sought to explore the relationship between SOB severity at discharge and subsequent complications. We aimed to identify a clinically relevant SOB threshold on the discharge day, serving as an early indicator of potential complications and enabling prompt medical intervention.

## Methods

### Study design and patients

Patients were selected from a dynamic perioperative rehabilitation cohort study of patients with lung cancer based on patient-reported outcome (PRO). The study enrolled patients undergoing treatment at Sichuan Cancer Hospital from April 2021 to November 2022. Eligibility criteria included: (1) undergoing lung surgery, (2) aged ≥ 18 years, and (3) having a pathological diagnosis of lung cancer. All participants provided informed consent. The cohort from April 2021 to January 2022 served as the development set, while the cohort from February 2022 to October 2022 functioned as the validation set. This cohort study has been approved by the Ethics Committee for Medical Research and New Medical Technology of Sichuan Cancer Hospital (No. SCCHEC-02-2018-043).

### Symptom measurements

The symptoms were assessed using the Perioperative Symptom Assessment for Lung Surgery (PSA-Lung) scale, a concise tool with validated reliability [17]. The PSA-Lung scale includes 7 symptom items and 2 functional items. Symptoms included pain, cough, SOB, fatigue, drowsiness, disturbed sleep, and distress; functional aspects included activity limitation and walking difficulty. The recall period was the last 24 h, during which patients rated the severity of the nine items on a 10-point scale, where “0” was no symptoms/no functional impairment and “10” was the worst symptom/functional impairment or dysfunction. Symptom data collection occurred preoperatively, daily during postoperative hospitalization, on the day of discharge, and daily for 1month post-discharge. Patients were monitored for postoperative complications after discharge. Data of Lung-PSA including the day of discharge and up to 1 month after discharge were analyzed in this study.

### Five-level EuroQol five-dimensional questionnaire

Five-level EuroQol five-dimensional questionnaire (EQ-5D-5 L) is a generic measure for assessing health-related quality of life [18]. Monthly follow-up assessments were conducted post-discharge using the EQ-5D-5 L questionnaire. Patients were asked to rate their current health status in the domains of mobility, pain/discomfort, self-care, usual activities, and anxiety/depression. The severity levels ranged from 1 (“no problems”) to 5 (“the worst problems”). Additionally, patients were requested to rate their overall health status, with “0” representing the worst health and “100” representing the best health. Data of EQ-5D-5 L collected one month after discharge was utilized in the current analysis.

### Postoperative complications after discharge

Complications occurring during and after surgery in patients are confirmed through relevant medical records and diagnostic data. Six experienced thoracic surgeons rotate to conduct telephone follow-ups to determine if patients experience complications within 90 days post-discharge. The diagnosis of all complications is based on the precise definitions set forth in the “Standardized Diagnosis and Treatment Terms for Thoracic Surgical Diseases.” The Clavien–Dindo classification criteria for surgical complications were used to score the severity of complications after surgery [19]. We defined the occurrence of complications as Clavien-Dindo grade ≥ I. If a thoracic surgeon is uncertain whether a case meets the diagnostic criteria for a complication, they consult with two other thoracic surgeons with over 5 years of experience to reach a consensus. All complication data and other relevant information are entered into a system and then verified by another staff member, who made corrections as necessary. This process was designed to ensure the highest level of data accuracy. Patient demographics, clinical information about the disease, and postoperative complications were captured and recorded in an electronic data management platform [20].

### Data analysis

Participants included in the analysis underwent at least one PSA-Lung assessment on the day of discharge or 1 day after discharge, and a minimum of three post-discharge PSA-Lung assessments per week for 1 month following discharge. For demographic and disease clinical information, normally distributed variables were described using the mean and standard deviation (SD) and t-test was used for between-group comparision; continuous variables that did not satisfy normal distribution were expressed in the form of median and interquartile range (IQR), with rank sum test for comparison; and categorical variables were expressed as the frequency and percentage, with the use of the χ2 test or Fisher’s exact test (when the number of digits in one of the cells was 5 or less).

Group-based trajectory modeling (GBTM) was utilized to describe the heterogeneity of SOB trajectories during the first month post-discharge. By combining the lowest Bayesian Information Criterion with clinical interpretability, a two-group model was identified, representing the low-symptom and high-symptom groups following hospital discharge.

Using the occurrence of complications 1 month after discharge as the dependent variable, a univariate analysis was performed with demographic information on patients’ age, sex, forced breathing, smoking history, tumor stage, duration of surgery, surgical approach, and SOB score on the day of discharge. Variables with a p-value < 0.05 were included in a multivariate logistic analysis to identify risk factors for the development of post-discharge complications.

To establish an early warning threshold for SOB at discharge, we used the occurrence of post-discharge complications as an anchor. We examined nine groups of different cutpoint (CP) that divided the symptom scale into two levels: CP1, CP2, CP3, CP4, CP5, CP6, CP7, CP8, and CP9. Logistic regression modeling was performed with post-discharge complications as the dependent variable and the nine sets of cutpoint as independent variables. We selected the cutpoint that generated the largest chi-square value as the optimal threshold for SOB, using the minimum P-value approach. To verify the robustness of the optimal cutpoint, we employed 2,000 samples of self-service resampling.

The optimal cutpoint identified in this study underwent external validation within the same patient cohort treated at Sichuan Cancer Hospital from February 2022 to October 2022. Patients were stratified into groups based on different SOB levels according to the cutpoint. Quality of life–including the comparison of the five dimensions: mobility, self-care, pain and anxiety, daily activities, and quality of life scores–was compared between the groups at 1 month post-discharge.

P value of less than 0.05 was considered statistically significant. All analysis processes are performed in SAS9.4 statistical analysis software.

## Results

### Patient characteristics

A total of 655 patients who met the criteria were included in this study. Table 1 presents the demographic and clinical details of the patients. The mean age was 54.39 ± 11.03, with females comprising the majority at 407 (62.14%). Most patients, 518 (79.08%), had never smoked, and 613 (93.59%) were diagnosed with early stage of lung cancer. Lobectomy was performed on approximately half of the patients, 359 (54.81%), and the vast majority, 606 (92.52%), received minimally invasive surgery.

**Table 1:** Demographics and Clinical Characteristics

Demographic or Clinical Characteristics	Total (*n* = 655)
Age (years), mean(SD)	54.39 (11.03)
FEV1 (L), median(IQR)	2.28 (1.72–2.83)
DLCO SB (mmol/min/kPa), median(IQR)	7.15 (5.79–8.68)
Postoperative length of stay in hostipal (days), median (IQR)	4 (3–6)
Operation time, median (IQR)	90 (65–120)
length of stay in hostipal (days), median (IQR)	8 (7–10)
Shortness of breath score on discharge, median (IQR)	4 (2–5)
Age	
≤55	345 (52.67%)
>55	310 (47.33%)
Sex	
Female	407 (62.14%)
Male	248 (37.86%)
ASA classification	
1	622 (94.96)
>1	33 (5.04%)
Smoking history	
No	518 (79.08%)
Yes	137 (20.92%)
Surgical approach	
Minimal invasive surgery^*^	606 (92.52%)
Open surgery	49 (7.48%)
Chest tube number	
One	519 (79.24%)
Two	136 (20.76%)
Extent of surgery	
Sub-lobectomy	290 (44.27%)
Lobectomy	359 (54.81%)
Others^**^	6 (0.92%)
Postoperative pathological TNM stage	
Early stage	613 (93.59%)
Locally advanced	42 (6.41%)

*Notes* Data are expressed as median (IQR) or n (%). *Minimal invasive surgery included 73 cases robotic-assisted thoracic surgery(RATS). **Others included 5 cases Pneumonectomy

Abbreviations: FEV1, forced expiratory volume in one second; DLCO SB, carbon monoxide diffusing capacity single-breath method; ASA, American Society of Anesthesiologists; TNM, tumor node metastasis; SD, standard deviation; IQR, 25th–75th percentile

### Post-discharge complications

A total of 71 patients (10.84%) in the study experienced complications within 3months post-discharge; of these, 65 (91.55%) occurred within 1 month after discharge, and 6 (8.45%) appeared between 1 and 3months post-discharge. Specifically, 23 patients (3.51%) developed postoperative pneumonia, 15 (2.29%) had pleural effusions, 11 (1.68%) experienced pneumothorax, and 23 (3.51%) experienced poor wound healing. The majority of post-discharge complications were concentrated within the first 2 weeks after discharge. Additionally, 21 patients (3.21%) encountered complications within 1month post-discharge, but the exact day of onset was not specified. Further details are provided in Additional file 1 (Table S1 and S2) .

**Table 2:** Multivariate Logistic Regression Analysis of Risk Factors for PCs after Hospital Discharge

	PCs(*n* = 71)	Non-PCs(*n* = 584)	Multivariate Analysis		
			OR	95%CI	*P*
Sex^*^					
Female	52 (40.60)	355 (59.40)	1.764	1.006–3.092	0.048
Male	19 (26.76)	229 (73.24)	Ref.		
Number of chest tube^*^					
Two	24 (17.65)	112 (82.35)	2.026	1.107–3.710	0.022
One	47 (9.06)	472 (90.94)	Ref.		
SOB score on the day of discharge^**^	5 (3–7)	4 (2–5)	1.125	1.012–1.250	0.030

*Notes* Statistically significant values are given in bold (*P* < 0.05); *N (%) ; **Median (IQR)

Abbreviations: PCs, Post-discharge complications; SOB, shortness of breath; OR, Odds Ratio; CI, confidence interval

### Identifying risk factors for post-discharge complications

Table S3 (see appendix) presents the univariate analysis. Female sex, duration of surgery, number of chest tube, and SOB score on the day of discharge were significantly associated with the occurrence of post-discharge complications. The results of the multivariate logistic regression analysis are shown in Table 2. Female sex (odds ratio [OR]: 1.764, 95% confidence interval [CI]: 1.006–3.092, *P* = 0.048), number of chest tube placements (OR: 0.494, 95% CI: 0.270–0.904, *P* = 0.022), and the SOB score on the day of discharge (OR: 1.125, 95% CI: 1.012–1.250, *P* = 0.030) were identified as significant risk factors for the development of post-discharge complications.

### GBTM-based analysis of SOB changes 1 month after discharge

**Fig. 1:**
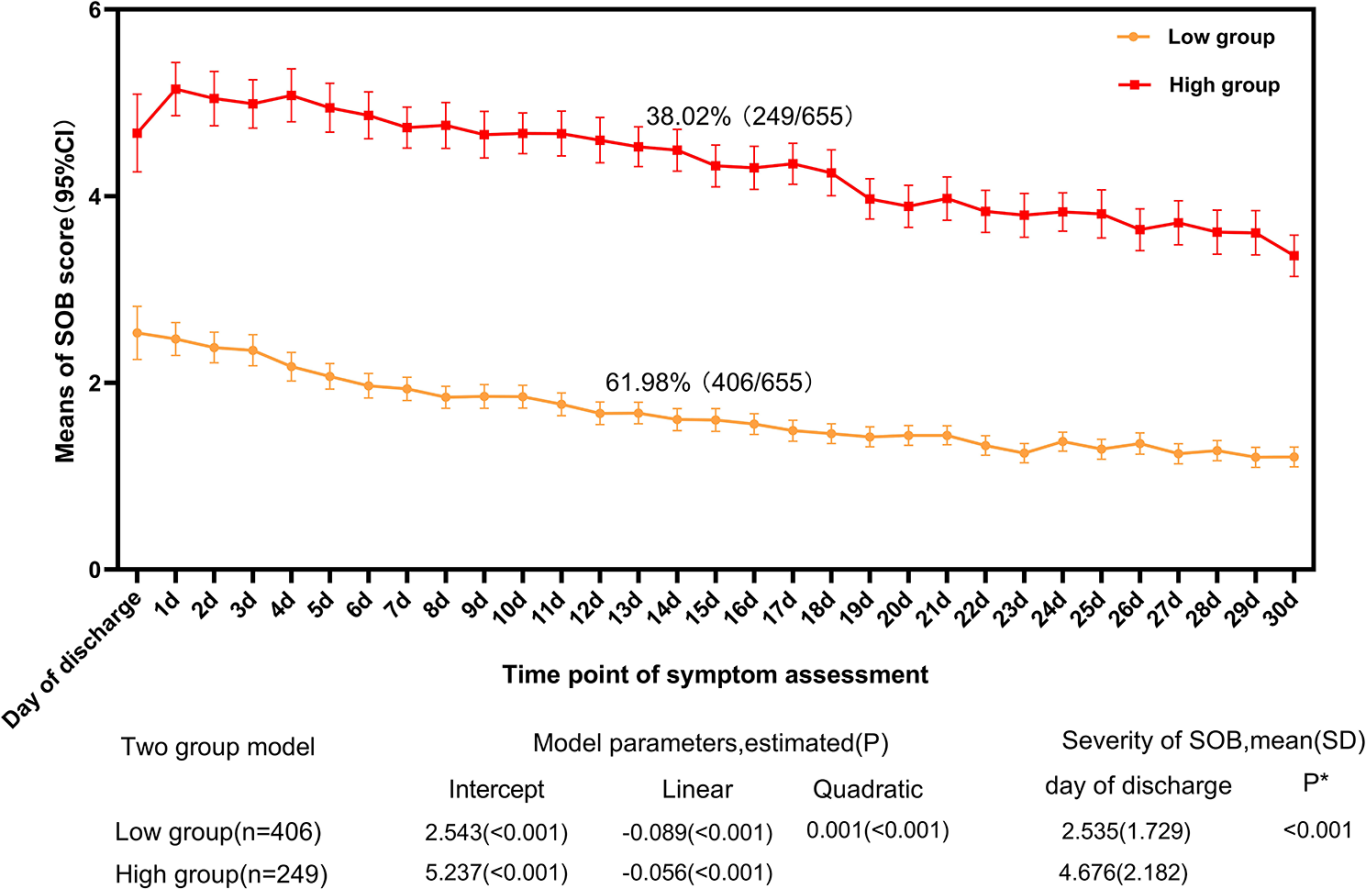
Trajectory for symptom of shortness of breath (SOB) in patients with lung cancer after surgery. *SOB severity in discharge between low and high symptom group by Wilcoxon rank-sum test

According to the results of GBTM plotted in Fig. 1, we defined the two-group symptom trajectories as patients-reported persistently with lower severity on SOB (61.98% of patients) or higher SOB (38.02% of patients) from the day of discharge to 1month post-discharge. In both models, the high-level symptom group exhibited significant linear and quadratic terms, while the low-level symptom group demonstrated only a significant linear trend (Fig. 1). The high-level symptom group presented moderate symptom values on the day of discharge (4.676 [2.182]), which increased and peaked on the first day post-discharge (5.146 [1.940])(Fig. 1). Conversely, the low-level symptom group experienced a mild peak in symptoms on the day of discharge (2.535 [1.729]), which subsequently decreased over time (Fig. 1). The Wilcoxon rank-sum test revealed a statistically significant difference in SOB symptom scores between the low-level and high-level symptom groups on the day of discharge (*P* < 0.001)(Fig. 1).

### Determining the optimal cutpoint for SOB

In the logistic regression model, the dependent variable was post-discharge complications, with nine different sets of cutpoint serving as independent variables. The highest Wald chi-square value was obtained at CP5. Table 3 presents the Wald chi-square values and P-values for the nine sets of cutpoint. To evaluate the robustness of the optimal cutpoint, self-help resampling was conducted with 2,000 samples (Table 4). Consequently, the optimal cutpoint for SOB on the first day post-discharge was established at 5, categorizing scores from 0 to 4 as the no/mild group and scores from 5 to 10 as the severe group.

**Table 3:** Optimal Cutpoint Analysis Using Anchor of Post-discharge Complications

cutpoint	Post-discharge complications	
	χ2 value	*P*
1	0.071	0.790
2	0.214	0.644
3	1.535	0.215
4	3.190	0.074
5	7.970	0.005
6	6.606	0.010
7	5.340	0.024
8	4.700	0.030
9	0.183	0.669

**Table 4:** Bootstrap with 2000 Resamplings for Cutpoint of shortness of breath

cutpoint	Post-discharge complications		
	χ2 value	95%CI	% as the Largest
5	2.221	2.103–2.339	57.91
6	0.128	0.087–0.169	42.09

### Validation of the SOB cutpoint

**Fig. 2:**
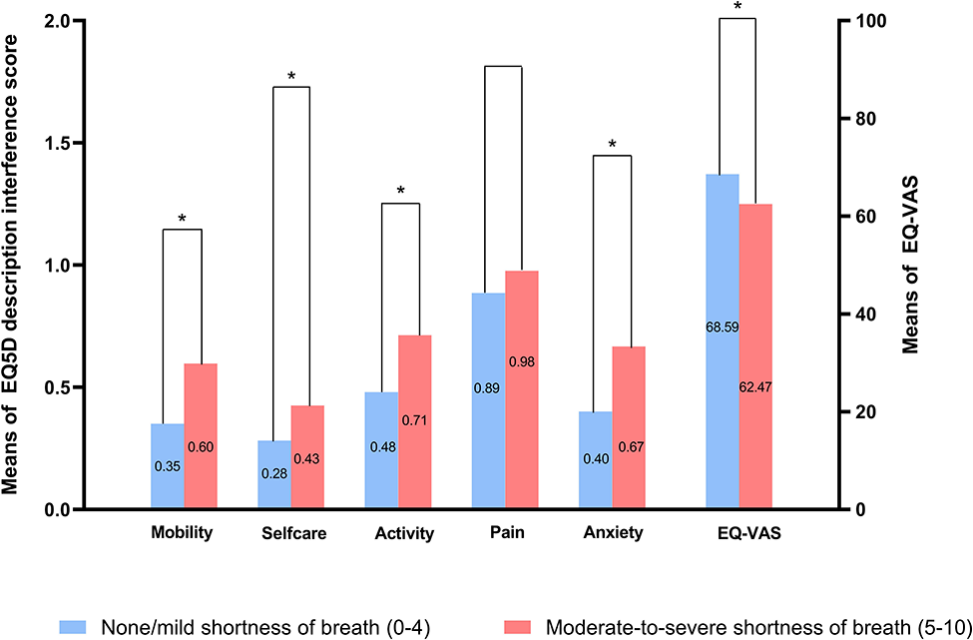
EQ-5D-5 L Quality of Life at different levels of shortness of breath (SOB) severity using the determined optimal cutpoint 5. * indicates statistical significance (*P* < 0.05)

Patients with an SOB score < 5 on the day of discharge reported fewer symptoms interfering with mobility [median (IQR), 0 (0–1) vs. 0 (0–1), *P* < 0.001], self-care [0 (0–0) vs. 0 (0–1), *P* = 0.008], daily activities [0 (0–1) vs. 1 (0–1), *P* = 0.002], anxiety or depression [0 (0–1) vs. 1 (0–1), *P* < 0.001], and reported better overall health [81 (65–91) vs. 73 (50–86), *P* = 0.006] 1 month after discharge (Fig. 2).

## Discussion

Our study may provide the evidence that being female and having two chest tubes, along with experiencing high symptom scores for SOB on the day of discharge, are significant risk factors for post-discharge complications. The significance of a high SOB symptom score on the day of discharge is underscored by the fact that being female and having two chest tubes may be non-modifiable factors for patients. Based on this finding, we determined that Patients who reported an SOB score of 5 or greater on the day of discharge were more likely to develop complications during the 3 month after discharge. And we used the occurrence of out-of-hospital complications as an anchor point, and a score of 5 (0–10) as the threshold for SOB at discharge, which was validated using patients’ quality of life reported at the end of the first month after discharge. Therefore, this threshold can be used as a warning signal for out-of-hospital complications.

Our study focused on monitoring and managing post-discharge complications, profiling their occurrence. The capture of post-discharge complications presents numerous challenges due to patients leaving the hospital environment. In such cases, standard medical records and clinical monitoring methods may not fully cover the patient’s actual health status, especially for non-acute or gradually developing complications. Therefore, we employed a variety of methods, including remote telephone follow-ups, conducted by experienced thoracic surgeons, to minimize data omissions and biases. Additionally, we implemented a strict data validation process, where any data regarding complications underwent consensus validation by at least two experts, ensuring data accuracy and reliability. These measures not only enhanced our understanding of postoperative complications but also improved the overall quality of the research.

Our findings on the incidence rate during the first month post-discharge are consistent with those reported by Cardinale et al. [21]. In the current era of early-stage lung cancer and minimally invasive surgery, sublobar resections and lobectomies constitute the vast majority, while total lung surgeries are exceedingly rare [22]. This is consistent with our study, where only 0.76% of patients underwent total lung resection. Consequently, the incidence of postoperative complications following total lung resection is also reduced [22–24], while for lobectomy, it ranges from 10 to 50% [25, 26]. Complications after pulmonary resection are classified as “early” or “late,” although the definitions for these categories vary by the specific complication [4]. Recent efforts to minimize postoperative complications include preoperative risk assessments, functional maneuverability assessments of patients, and prediction of risk factors [27].Clearly, preoperative risk assessment and risk factor prediction are essential for identifying high-risk patients and reducing the likelihood of complications. Numerous recent studies have shown a persistent high incidence of post-discharge complications, aligning with our findings. However, it is important to note that many studies fail to include these results as parameters [14]. In addition, Despite our efforts to minimize biases and errors, we must cautiously consider that the monitoring of post-discharge complications may still be limited by patients’ reporting and tracking difficulties.Therefore, future research needs to explore more technologies and strategies, such as digital health tools and real-time data monitoring, to further improve the quality and integrity of postoperative complication data collection, thereby maximizing its value as a clinical outcome parameter.

Age, Charlson comorbidity index, surgical approach, and smoking history have been identified as risk factors for postoperative complications in patients undergoing pneumonectomy [27–31]. These risk factors enable clinicians to assess the level of risk for complications and to intervene proactively [32, 33]. Unlike our previous study [16], the fact that being female was identified as a risk factor for post-discharge complications in patients who underwent lung surgery in this study is consistent with the study of Brian M. Lin et al. [34] who also found that females were associated with a higher risk of postoperative complications in their study. This may be attributed to the fact that females often play the role of caregivers within the family [34]. Additionally, our study identified the number of chest tube placement roots and the SOB symptom score on the day of discharge as risk factors for postoperative out-of-hospital complications in patients with lung cancer. While pre-discharge complications have been extensively reported, post-discharge complications have been less studied [35]. We hypothesize that the inconsistency in risk factor prediction between this study and others may be because our analysis is more suited to predicting out-of-hospital complications. Preoperative patient risk assessment and predicted risk factors are effective for identifying complications during hospitalization but may not be as indicative of post-discharge complications.

The importance of symptom monitoring extends beyond providing information for symptom assessment. It also includes other relevant aspects, such as the use of symptom cutpoint as alarm thresholds [36]. Thresholds have been widely developed and utilized in clinical practice and medical guidelines to enhance patient-provider communication, assess treatment outcomes, and guide clinical decision-making [36]. Among these, the establishment of thresholds is often recommended to be based on anchor-based methods, ensuring clinical significance and interpretability [37]. However, when selecting anchor points, the majority of studies have primarily focused on PRO scores related to interference with daily functioning and verbal ratings of symptom severity [36]. Although postoperative complications are crucial indicators for patient recovery, there has been limited research using them as an anchor for establishing cutpoint to aid clinical decision-making. In our study, choosing post-discharge complications as the anchor provided a clinically meaningful approach to using PROs in the identification of high-risk individuals, development of management strategies, and promotion of patient prognosis, aligning with findings from oncology practice [38].

Cleeland et al. demonstrated that out-of-hospital symptom monitoring, enabling timely warning and feedback, leads to better control and reduces emergencies [39]. Postoperative complications have been extensively studied during hospitalization, but little is known about them once the patient is discharged [35]. Earlier studies have provided a practical and interpretable tool for identifying patients at risk of developing complications during postoperative hospitalization, facilitating proactive prevention and control measures within the hospital setting [16]. Our current study enhances the previous work by adding early warning alerts for SOB related to out-of-hospital complications in discharged patients. This enables timely monitoring of PROs outside the hospital, offering a valuable tool for identifying post-surgical patients at risk after discharge. Our analysis revealed associations between SOB and complications, functional impairment, and quality of life after discharge. Clinicians can use a simple SOB score, with a clinically interpretable threshold, to evaluate a patient’s status and determine whether an extensive care plan should be applied for the patient at home upon discharge.

The current findings suggest that implementing an extensive care strategy for patients with an SOB score ≥ 5 on the day of discharge could be beneficial. Healthcare providers can inform these patients about the implications of a higher SOB score, recommend appropriate home care measures, manage symptoms, support recovery, and implement a more rigorous follow-up schedule. It may be beneficial to assign dedicated outpatient care coordinators to this subgroup of patients to closely monitor their symptoms for timely intervention and to reduce the risk of complications. We have evidenced via RCTs that utlizing electronic PRO-based (ePRO) symptom monitoring via smartphone during the first month after discharge could enhance recovery and lower complications in patients after lung cancer surgery, compared with the usual care [40].

One limitation of this study is the established threshold of SOB ≥ 5, which was based on data from Chinese patients discharged after lung cancer surgery. This threshold may not be universally applicable, as patients with different diseases or from various cultural backgrounds may exhibit distinct physical and psychological characteristics. Consequently, further research and validation are required to confirm the threshold’s applicability and accuracy before it can be applied to other populations. Moreover, the study relied solely on symptom assessment at the time of discharge to establish thresholds. However, the prediction of post-discharge complications might necessitate consideration of time points beyond the day of discharge, indicating the need for more sensitive selection criteria. Future studies should consider designing a multi-timepoint, comprehensive threshold to improve sensitivity. Additionally, it is crucial to meticulously document any reasons for a patient’s non-cooperation with the follow-up visit on that day to ensure data completeness and accuracy. Such diligence will enhance the reliability of the study’s findings and foster a more thorough understanding of the patient’s symptomatology during the postoperative recovery period.

## Conclusion

In conclusion, our study identified being female, having two chest tubes, and high SOB symptom scores on the day of discharge as risk factors for complications within 3 months after discharge. By monitoring discharged patients’ SOB and establishing symptom thresholds, we may predict the risk of post-discharge complications. This approach enables the identification of patients in need of comprehensive care, potentially reducing unplanned readmissions and alleviating the long-term disease burden.

### Electronic supplementary material

Below is the link to the electronic supplementary material.

Supplementary Material 1

## Data Availability

The datasets generated during and/or analyzed during the current study are available from the corresponding author on reasonable request.

## Abbreviations

PRO: Patient-reported outcome
SOB: Shortness of breath
PSA: Lung-Perioperative Symptom Assessment Scale for Lung surgery
EQ: 5D-5 L-Five-level EuroQol five-dimensional questionnaire
RATS: Robotic-assisted thoracic surgery
SD: Standard deviation
IQR: 25th-75th percentile
GBTM: Group-based trajectory modeling
CP: Cutpoint
FEV1: Forced expiratory volume in one second
DLCO SB: Carbon monoxide diffusing capacity single-breath method
ASA: American Society of Anesthesiologists; TNM: tumor node metastasis
OR: Odds Ratio
CI: Confidence interval
