# Supplementary material for: Shortness of breath on the day of discharge: an early alert for post-discharge complications in patients undergoing lung cancer surgery
Source: J Cardiothorac Surg. 2024 Jun 27;19:398. doi: 10.1186/s13019-024-02845-1 (PMC11210099; doi:10.1186/s13019-024-02845-1)
Supplement: Supplementary file 1 — Supplementary Material 1 [file 13019_2024_2845_MOESM1_ESM.docx]

**Table S1 Details of the time when complications occurred after discharge（N=71）**

| Post-discharge complications（n=71） | Frequency | Rate% |
| --- | --- | --- |
| At discharge | 1 | 0.15 |
| 1 day after discharge | 1 | 0.15 |
| 2 days after discharge | 2 | 0.31 |
| 3 days after discharge | 3 | 0.46 |
| 4 days after discharge | 5 | 0.76 |
| 5 days after discharge | 1 | 0.15 |
| 6 days after discharge | 2 | 0.31 |
| 7 days after discharge | 3 | 0.46 |
| 8 days after discharge | 1 | 0.15 |
| 9 days after discharge | 3 | 0.46 |
| 10 days after discharge | 1 | 0.15 |
| 11 days after discharge | 3 | 0.46 |
| 12 days after discharge | 2 | 0.31 |
| 13 days after discharge | 0 | 0.00 |
| 14 days after discharge | 2 | 0.31 |
| 15 days after discharge | 1 | 0.15 |
| 16 days after discharge | 2 | 0.31 |
| 17 days after discharge | 0 | 0.00 |
| 18 days after discharge | 2 | 0.31 |
| 19 days after discharge | 2 | 0.31 |
| 20 days after discharge | 0 | 0.00 |
| 21 days after discharge | 1 | 0.15 |
| 22 days after discharge | 1 | 0.15 |
| 23 days after discharge | 0 | 0.00 |
| 24 days after discharge | 1 | 0.15 |
| 25 days after discharge | 1 | 0.15 |
| 26 days after discharge | 1 | 0.15 |
| 27 days after discharge | 0 | 0.00 |
| 28 days after discharge | 0 | 0.00 |
| 29 days after discharge | 0 | 0.00 |
| 30 days after discharge | 2 | 0.31 |
| Within 1 month of discharge^*^ | 21 | 3.21 |
| 1-3 months after discharge | 6 | 0.92 |
| Overall morbidity | 71 | 10.84 |

**Note: *Only this portion of the population could be determined to have occurred within 30 days of discharge from the hospital**

**Table S2 Details of post-discharge complications （N=655）**

| Post-discharge complications（N=71） | Frequency | Rate% | Clavien-Dindo grading | | | | |
| --- | --- | --- | --- | --- | --- | --- | --- |
|  |  |  | I | II | III | IV | V |
| Postoperative pneumonia | 23 | 3.51 |  | 23 |  |  |  |
| Postoperative pleural effusion | 15 | 2.29 |  |  | 15 |  |  |
| Postoperative pneumothorax | 11 | 1.68 |  |  | 11 |  |  |
| Postoperative arrhythmia | 1 | 0.15 |  | 1 |  |  |  |
| Heart failure | 1 | 0.15 |  |  |  | 1 |  |
| Hoarseness | 1 | 0.15 | 1 |  |  |  |  |
| Postoperative hemoptysis | 2 | 0.31 |  | 2 |  |  |  |
| Gastric emptying disorder | 1 | 0.15 |  |  | 1 |  |  |
| Wound infection | 6 | 0.92 | 6 |  |  |  |  |
| Incision dehiscence/poorly healed | 23 | 3.51 | 23 |  |  |  |  |
| Subcutaneous emphysema | 5 | 0.76 |  |  | 5 |  |  |
| Others | 2 | 0.31 | 2 |  |  |  |  |
| Overall morbidity | 71 | 10.84 |  |  |  |  |  |

**Note: Two types of complications were reported in 16 patients;**

**Three types of complications were reported in 1 patients;**

**Four types of complications were reported in 1 patients.**

Table S3 Univariate Analysis of Risk Factors for Post-discharge Complications

|  | PCs（n=71） | Non-PCs（n=584） | *P* |
| --- | --- | --- | --- |
| Age |  |  | 0.546 |
| ≤55 | 35（49.30） | 310（53.08） |  |
| ＞55 | 36（50.70） | 274（46.92） |  |
| Sex |  |  | **0.041** |
| Male | 19（26.76） | 229（39.21） |  |
| Female | 52（73.24） | 355（60.79） |  |
| FFEV1 | 2.18（1.56-2.60） | 2.30（1.74-2.85） | 0.137 |
| DLCOSB | 6.87（5.57-8.13） | 7.20（5.83-8.74） | 0.201 |
| Operation time | 100（80-130） | 90（65-120） | **0.015** |
| Postoperative length of stay in hospital | 4（3-6） | 4（3-5） | 0.427 |
| Surgical approach |  |  | 0.419 |
| Minimal invasive surgery | 64（90.14） | 542（92.81） |  |
| Open surgery | 7（9.86） | 42（7.19） |  |
| Chest tube number |  |  | **0.004** |
| One | 47（66.20） | 472（80.82） |  |
| Two | 24（33.80） | 112（19.18） |  |
| Extent of surgery |  |  | 0.853 |
| Sub-lobectomy | 30（42.25） | 260（44.52） |  |
| Lobectomy | 40（56.34） | 319（54.62） |  |
| Other | 1（1.41） | 5（0.86） |  |
| ASA |  |  | 0.226 |
| 1 | 68（75.77） | 554（94.86） |  |
| ＞1 | 3（4.23） | 30（5.14） |  |
| Smoking history |  |  | 0.567 |
| No | 58（81.69） | 460（78.77） |  |
| Yes | 13（18.31） | 124（21.23） |  |
| Postoperative pathologic TNM stage |  |  | 0.441 |
| Early stage | 65（91.55） | 548（93.84） |  |
| Locally advanced | 6（8.45） | 36（6.16） |  |
| Shortness of breath score the day of  discharge | 4.61（2.52） | 3.86（2.50） | **0.010** |

Notes: Statistically significant values are given in bold （P< 0.05）;
